# Supplementary material for: Fermentation Extract of Naringenin Increases the Expression of Estrogenic Receptor β and Modulates Genes Related to the p53 Signalling Pathway, miR-200c and miR-141 in Human Colon Cancer Cells Exposed to BPA
Source: Molecules. 2022 Oct 5;27(19):6588. doi: 10.3390/molecules27196588 (PMC9572342; doi:10.3390/molecules27196588)
Supplement: Supplementary file 1 [file molecules-27-06588-s001.zip › molecules-1915724-supplementary.pdf]

SUPPLEMENTARY MATERIAL

Table S1. Forward and reverse sequences of genes REα, REβ, DNMT1 and GPER30.

Gene	FWD-PRIMER	REV-PRIMER	Alignment Tm
β-actin	5´-ACGGGGTCACCCACACTGTGC-3´	CTAGAAGCATTTGCGGTGGACGATG	62°C
ERβ	5´-TCCCACTTCGTAACACTTCCG-3´	ACATTCTATAGCCCTGCTGTGA	64°C
GPR30	5´-AGTCGGATGTGAGGTTCAG-3´	TCTGTGTGAGGAGTGCAAG	60°C

Table S2. Stem Loop sequences and specific primer of miRNA 200c and 141, universal antisense and Forward and Reverse sequence of U6.

Primer	Sequence
Stem Loop miR-200c	5' –GTTGGCTCTGGTGCAGGGTCCGAGGTAT TCGCACCAGAGCCAACTCCA TC - 3'
Stem Loop miR-141	5' –GTTGGCTCTGGTGCAGGGTCCGAGGTAT TCGCACCAGAGCCAACCCATCT - 3'
Universal antisense	5' - GTGCAGGGTCCGAGGT - 3'
Specific initiator miR-200c	5' - TGGGTTAATACTGCCGGGTAAT - 3'
Specific initiator miR-141	5' - GGGGGTAACACTGTCTGGTAA - 3'
REV U6	5'- AAAATATGGAACGCTTCACGAAT – 3'
FWD U6	5'- CGCTTCGGCAGCACATATACTA – 3'
